# Correction: Psychosocial and socio-environmental factors associated with adolescents’ tobacco and other substance use in Bangladesh

**DOI:** 10.1371/journal.pone.0253672

**Published:** 2021-06-21

**Authors:** Md. Mostaured Ali Khan, Md. Mosfequr Rahman, Syeda S. Jesmin, Md. Golam Mustagir, Md. Rajwanul Haque, Md. Sharif Kaikobad

The third author’s name is spelled incorrectly. The correct name is: Syeda S. Jesmin. The correct citation is: Khan MMA, Rahman MM, Jesmin SS, Mustagir MG, Haque MR, Kaikobad MS (2020) Psychosocial and socio-environmental factors associated with adolescents’ tobacco and other substance use in Bangladesh. PLoS ONE 15(11): e0242872. https://doi.org/10.1371/journal.pone.0242872
