# Supplementary material for: Psychosocial and socio-environmental factors associated with adolescents’ tobacco and other substance use in Bangladesh
Source: PLoS One. 2020 Nov 24;15(11):e0242872. doi: 10.1371/journal.pone.0242872 (PMC7685447; doi:10.1371/journal.pone.0242872)
Supplement: Attachment — Submitted filename: Response to Reviewers.docx [file pone.0242872.s001.docx]

September 8, 2020

Prof. Stanton A. Glantz
Academic Editor
PLOS ONE

**Re: Manuscript Number PONE-D-20-18742**

Dear Prof. Stanton A. Glantz,

Thank you very much for your kind consideration of our manuscript entitled, ***“Psychosocial and socio-environmental factors associated with adolescents’ tobacco and substance use in Bangladesh”.*** We have revised the manuscript according to the journal’s requirements, editor’s comments and to the comments by the reviewers. Their useful and productive comments helped us to improve the clarity and quality of the manuscript. Where we have changed the text, the corresponding sentences in the text have been highlighted with the track changes function. The page and line numbers referred to are those in the margins of our clean revised manuscript. Where textual changes are included in this response to reviewer, we have indented and italicized them, and removed references.

It is important to mention here that this study did not receive grant from any funding agency. Unfortunately, we provided the following statement while submitting the manuscript initially: **The funders had no role in study design, data collection and analysis, decision to publish, or preparation of the manuscript.** Since we did not receive any grant please fill-up the appropriate option for that.

We hope that the revisions are satisfactory in addressing the issues raised by the editor and the reviewers, and look forward to hearing your decision about this article.

Yours sincerely,

Md. Mosfequr Rahman, PhD

Department of Population Science and Human Resource Development

University of Rajshahi

Rajshahi-6205, Bangladesh.

E-mail: [mosfeque@ru.ac.bd](mailto:mosfeque@ru.ac.bd)

**Journal Requirements:**

1. Please ensure that your manuscript meets PLOS ONE's style requirements, including those for file naming. The PLOS ONE style templates can be found at

<https://journals.plos.org/plosone/s/file?id=wjVg/PLOSOne_formatting_sample_main_body.pdf> and

<https://journals.plos.org/plosone/s/file?id=ba62/PLOSOne_formatting_sample_title_authors_affiliations.pdf>

**Response**: Thank you. We have revised the manuscript according the guidelines of PLOS ONE.

2.We suggest you thoroughly copyedit your manuscript for language usage, spelling, and grammar. If you do not know anyone who can help you do this, you may wish to consider employing a professional scientific editing service.  

Whilst you may use any professional scientific editing service of your choice, PLOS has partnered with both American Journal Experts (AJE) and Editage to provide discounted services to PLOS authors. Both organizations have experience helping authors meet PLOS guidelines and can provide language editing, translation, manuscript formatting, and figure formatting to ensure your manuscript meets our submission guidelines. To take advantage of our partnership with AJE, visit the AJE website (http://learn.aje.com/plos/) for a 15% discount off AJE services. To take advantage of our partnership with Editage, visit the Editage website ([www.editage.com](http://www.editage.com/)) and enter referral code PLOSEDIT for a 15% discount off Editage services.  If the PLOS editorial team finds any language issues in text that either AJE or Editage has edited, the service provider will re-edit the text for free.

 Upon resubmission, please provide the following:

- The name of the colleague or the details of the professional service that edited your manuscript
- A copy of your manuscript showing your changes by either highlighting them or using track changes (uploaded as a *supporting information* file)
- A clean copy of the edited manuscript (uploaded as the new *manuscript* file)

**Response:** Thank you. This manuscript has been copyedited thoroughly for language usage, spelling, and grammar by one of our co-author Syeda S. Jesmin, who is an Associate Professor of Sociology and Psychology, University of North Texas at Dallas, Dallas, TX, USA. She can be contacted by the following e-mail: [syeda.Jesmin@untdallas.edu](mailto:syeda.Jesmin@untdallas.edu).

3.Thank you for stating the following financial disclosure:

 [The funders had no role in study design, data collection and analysis, decision to publish, or preparation of the manuscript.].

At this time, please address the following queries:

a. Please clarify the sources of funding (financial or material support) for your study. List the grants or organizations that supported your study, including funding received from your institution.
b. State what role the funders took in the study. If the funders had no role in your study, please state: “The funders had no role in study design, data collection and analysis, decision to publish, or preparation of the manuscript.”
c. If any authors received a salary from any of your funders, please state which authors and which funders.
d. If you did not receive any funding for this study, please state: “The authors received no specific funding for this work.”

 Please include your amended statements within your cover letter; we will change the online submission form on your behalf.

**Response:** The authors received no specific funding for this work.

**Reviewer #1**

This is a study using on the psychosocial and socio-environmental factors associated with tobacco use and substance use (combined alcohol and/or marijuana use). They find a dose-response relationship between adverse psychosocial factors and tobacco use. The likelihood of substance use (alcohol and/or marijuana) was higher among adolescents who experienced 3+ adverse psychosocial events. Tobacco and substance use in an adjusted logistic analysis, those who were bullied, and had ‘adverse sexual history’ whatever that means. Substance use is associated was associated with loneliness, lack of peer support and higher. Tobacco use was associated with anxiety-related sleep loss parental tobacco or drug use, poor parental understanding, poor parental monitoring and lack of peer support.

This study is an important update of data from 2014 to the previously published papers on adolescent tobacco and substance use using the 2007 data from the Global School-based Student Health Survey for Bangladesh.

**Response:** Thank you for your appreciations.

1. You must say where the publicly available database is located and how to access it

**Response:** Thank you very much. We have provided the following links in the text for the location of GSHS data (Page 7; Lines: 143-144).

A detailed description of the GSHS and data used in this study are available at: <https://extranet.who.int/ncdsmicrodata/index.php/catalog/485> and <https://www.cdc.gov/gshs/countries/seasian/bangladesh.htm>

2. CDC is Centers for Disease Control and Prevention

**Response:** Yes, it is. We have corrected it in the manuscript.

3. Explain what you mean by adverse sexual history. If it means yes = have had sex, no = have not had sex, then you should call the variable sexual history or something less loaded than adverse sexual history. I see that you do that in Table 1. Call the variable sexual history or some other less loaded term throughout the text

**Response:** Thank you. We called this variable as “sexual history” and corrected accordingly throughout the text and tables.

4. Line 247: male adolescents are less monitored or supervised.

**Response:** We have revised it accordingly.

5. Table 3: Physical abused should be physically abused

**Response:** We have corrected it accordingly.

6. Explain why you decided to lump together alcohol and marijuana use into substance use. For a general reader from a non-Muslim majority country, this may seem strange

**Response:** Thank you very much. We have added alcohol and marijuana use together and named the variables as “substance use”, because both of these substances are illegal in Bangladesh. Smoking cigarette is not illegal but against the cultural norms and also unacceptable. We wanted to differentiate these two. The following sentence is added to the text for clarifying it (Page 8, Lines 153-154):

*“Substance use (SU) was assessed combining the use of two illegal substances- alcohol and marijuana”*

7. Table 2: the subscripts that you give to the table – a) in past 12 months, b) in past 30 days – are never used in Table 2.

**Response:** Thank you. We are sorry for such unintentional mistakes. We have corrected it in Table 2.

**Reviewer #2**

Overall, this is an important study that examines adolescent substance use and tobacco use, and underlying factors of such use among adolescents in Bangladesh. The manuscript would benefit from a more in-depth description of the problem and context. The manuscript is lacking several aspects of the literature, importantly, the gaps that the study data fill and how the approach in this study will help future research, policy and practice. Since the data are from 2014, the authors may need to justify why they sought to study prevalence when more recent data have already been published from 2017-18. Several aspects of the methods could include greater detail and justification. The study would also do well to include more details in the Discussion based on comparisons with other low-and middle-income countries or other similar socio-religious contexts. Specifically, the authors should modify their conclusion that this study provides information about predictors of tobacco and substance use as it is a cross-sectional study and not a longitudinal one.

**Response:** Thank you very much. We have addressed the issues one by one in the following sections.

**Abstract**

The authors may want to be consistent in saying use/ misuse/ abuse.

**Response:** Thank you very much. We have used ‘use’ consistently in the abstract and text, and corrected accordingly.

At the outset in the abstract, it is unclear as to how the authors’ have used a cross-sectional study design to ascertain predictors of tobacco and substance use. The description of results indicates that the authors have assessed associations/ relationships between such use (TU and SU) and factors. In multiple places the authors have stated that they determined predictors, which is not feasible using this study design.

**Response:** Thank you. Yes, predictors should not be used in cross-sectional study design. We have corrected it throughout the manuscript where we use predictors.

Provide Odds Ratios for: “Experience of adverse socio-environmental factors, such as parental substance use, poor monitoring and understanding, and lack of peer support were also positively associated with TU and/or SU.”

**Response:** We have provided odds ratios in abstract in our revised manuscript.

It is not clear what the authors mean by: “Additionally, multiple adverse psychosocial factors were associated with TU in a graded manner.”

**Response:** While we examined the relationship between adolescents’ multiple adverse experiences with TU and SU, we observed a graded relationship between psychosocial factors and TU. This means that the odds of TU are gradually increasing with the increment of multiple experiences of adverse psychosocial events. We have corrected the sentence as follows for clear understanding (Page 3, Lines 45-46).

*“Additionally, adolescents’ multiple adverse experience of psychosocial factors was found to be associated with TU in a graded manner.”*

It is not clear what the authors mean by: “Tobacco and substance use among school-going adolescents in Bangladesh are relatively prevalent.” Relative to what percentage, what age group, and where?

**Response:** Thank you. The prevalence TU and SU that we found in our study was much lower than the prevalence found in some other developing countries like Ethiopia, Ghana, Philippines, Namibia, Chili etc (References given below). These studies were also used the data of respective country’s GSHS. Therefore, we wrote relatively. Since it creates confusion, we deleted the term ‘relatively’ from the sentence.

Page, R.M., et al., *Psychosocial distress and substance use among adolescents in four countries: Philippines, China, Chile, and Namibia.* Youth & society, 2011. **43**(3): p. 900-930.

Pengpid, S. and K. Peltzer, *Alcohol use and misuse among school-going adolescents in Thailand: results of a national survey in 2015.* International journal of environmental research and public health, 2019. **16**(11): p. 1898

Birhanu, A.M., T.A. Bisetegn, and S.M. Woldeyohannes, *High prevalence of substance use and associated factors among high school adolescents in Woreta Town, Northwest Ethiopia: multi-domain factor analysis.* BMC public health, 2014. **14**(1): p. 1186.

Hormenu, T., et al., *Psychosocial Determinants of Marijuana Utilization Among Selected Junior High School Students in the Central Region of Ghana.* Journal of Preventive Medicine and Care, 2018. **2**(2): p. 43.

Why was “substance use” created as an outcome measure? Why did the authors not use any other validated measure on substance use?

**Response:** Thank you. Alcohol, marijuana, and tobacco are substances most commonly used by adolescents (References below).

Johnson LD, O’Malley PM, Bachman JG, Schulenberg JE, Miech RA. Monitoring the Future national survey results on drug use, 1975-2013: Volume 1, Secondary school students. Ann Arbor, MI: Institute for Social Research, University of Michigan, 2014: 32-36.

Latimer, W. and J. Zur, Epidemiologic trends of adolescent use of alcohol, tobacco, and other drugs. Child and Adolescent Psychiatric Clinics, 2010. 19(3): p. 451-464.

World Drug Report 2018 (United Nations publication, Sales No. E.18.XI.9)

Ramo, D.E., H. Liu, and J.J. Prochaska, *Tobacco and marijuana use among adolescents and young adults: a systematic review of their co-use.* Clinical psychology review, 2012. **32**(2): p. 105-121.

We could use separate variables for alcohol drinking and marijuana use, however, alcohol drinking and marijuana use is strictly prohibited by law in Bangladesh. So information on these is rare. Therefore, we have combined these two and named it ‘substance use’. Since we want to assess how psychosocial and environmental factors are associated with adolescents’ substances use (smoking, alcohol drinking and marijuana use), therefore we use these as outcomes.

What forms of marijuana use were included?, since ‘drinking’ alcohol is specified, it would be good to maintain parallelism in the sentence.

**Response:** Thank you. Yes it would be nice to have similar structure of the questionnaire. However, we used a nationally representative secondary data (GSHS), so we have to use the questionnaire and the data as they collected. The questionnaire of the GSHS can be found in the following link:

<https://www.cdc.gov/gshs/questionnaire/index.htm>

**Introduction**

If it is known that 6.9% of adolescents are tobacco users, and 2% are alcohol users from 2017-2018 data, which is based on more recent data than the study under review, what is the justification for this study based on data from 2014? What is the gap that the current study fills in terms of assessing prevalence?

**Response:** Thank you for your query. Actually, the references (4 and 5, in earlier version of the manuscript) used for documenting the prevalence were published in 2018 and 2017; but they used data from Global Youth Tobacco Survey (GYTS), Bangladesh 2013, and the WHO STEPwise Surveillance (STEPS) data in 2010, respectively. And this current analysis is based on data collected in 2014, therefore, the prevalence reported in this current study is from the latest data. However, to avoid confusion we have rewritten the sentence as follows and used the original references:

*“The 2013 Global Youth Tobacco Survey of Bangladesh reported that 6.9% of adolescents (9.2% males and 2.8% females) are currently tobacco users. The prevalence of alcohol use across age groups is less than one percent (0.8%; men 1.5% and women 0.1%.”*

Suggestion to include a description of the types of tobacco use in Bangladesh, as there may be many diverse forms of use. Line 99 – “limited to only tobacco (smoke or smokeless)”

**Response:** Thank you. We have added the following sentence in the first paragraph describing types of tobacco use in Bangladesh (Page 4, Lines 59-61)

*“Around 35% of the total population in Bangladesh use either smoked cigarettes or bidis or smokeless tobacco products (tobacco flakes known as Zarda, betel leaf quid or paan, and gul”*

What makes the authors believe that the reasons for adolescents using tobacco in global studies differ from the reasons why adolescents use in Bangladesh?

**Response:** Although there are some restrictions of smoking in public places and selling cigarettes to the minors; tobacco smoking or using other forms of tobacco is not prohibited by law in Bangladesh. Like many other countries, using tobacco, specifically smoking among adolescents is also socially and culturally unacceptable in Bangladeshi. The numbers of tobacco smokers are increasing rapidly because of the availability of cheap tobacco products, lack of strong tobacco control regulations, and weak enforcement of existing regulations. However, alcohol drinking and using marijuana is prohibited by law in Bangladesh. Since using these substances has enormous health impact among the adolescent, therefore more studies are required to identify whether the same factors contributes to substances use in Bangladesh and in other settings. Factors those are found to be associated with tobacco use in global studies might be true for Bangladesh. However, if data is available, it would be better to find out whether these factors that are actually associated or not with respective country’s data. It would help formulate appropriate culturally acceptable interventions. That’s why our study is important and has implication for Bangladesh.

Line 98-99 What is the reason why the authors want to include an urban sample on tobacco users? What proportion of Bangladesh is urban?

**Response:** Thank you. The proportion of urban population in Bangladesh is around 37% (According to statista in 2018; link below). Some earlier studies in Bangladesh did not use country representative sample, rather used only rural sample. Therefore, it is not possible to generalize the findings to the whole country. To get the whole picture of substance use, we need country representative data, not partial one. We, in our study, fill that gap by identifying factors associated with substance use using nationally representative sample which represents the whole country.

https://www.statista.com/statistics/761021/share-of-urban-population-bangladesh/

Line 100: What is the evidence that psychosocial factors are important? Perhaps the authors would like to define these factors, explain briefly how they are likely interlinked. In the paragraph on global studies (line 78-89) the authors mention many factors, but latel them interpersonal risk factors, protective factors, psychosocial distress, and so one is not sure what psychosocial factors entail exactly?

**Response:** Thank you. For addressing this issue we have added the following sentences in the text (Page 5 Line 92-101).

*“There is a large body of literature that highlights psychosocial factors such as, loneliness, stress, depression, anxiety, hyperactivity/inattention, and low self-esteem as risk factors for substance abuse. For example, Page et. al. reported in a four country study (Philippines, China, Chile, and Namibia) that among adolescents often feeling lonely, worried, sad/hopeless, or having a suicide plan were associated with current smoking and drinking alcohol. Findings from a longitudinal study in Australia suggest that psychosocial risk factors, such as truancy, hyperactivity/inattention, and behavior problems, are associated with adolescent binge drinking and cannabis use. A recent review concludes that psychosocial risk factors of adolescent substance use are closely related to peer influence, and that parents tend to have the strongest effect on adolescents’ substance use behavior”*

Line 106: MAE – Multiple adverse experiences – This construct is mentioned without any citation or background information. It seems like it is better suited as a measure rather than mentioned in passing at the end of the introduction.

**Response:** We have added following sentence in the introduction section justifying the inclusion of MAE here (Page 6 Lines 116-118).

*“A growing number of studies document a graded relationship between adverse life circumstances and negative health outcomes, including health risk behaviors, such as substance use. Therefore, we examine to what extent multiple adverse experiences (MAE) affect tobacco and substance use behaviors among the adolescent population.”*

We also have discussed briefly about the calculation procedure of MAE scores in our methodology section. Following paragraph is added to the text (Page 8-9, Lines 165-174)

***“Calculation of multiple adverse experience (MAE) scores***

*We followed the Kaiser Permanente Childhood Adverse Experience (ACE) study to calculate the multiple adverse experience (MAE) score. We divided adolescents’ adverse experiences into two categories: psychosocial and socio-environmental factors. Details of these two categories are presented in Table 1. The MAE score for each adverse category was calculated by summing up the responses on the adverse experience questions. For each adverse experience, the response 0 meant the participant never or rarely experienced the adversity, while 1 meant the participant always or sometimes experienced that specific adversity. Higher MAE meant greater experience of adverse events. As for example, if an individual has experienced three adverse events, then the MAE score is 3.”*

**Data and Methods**

Line 119: What is the disadvantage of using a school-based sample?

**Response:** Thank you. The GSHS was conducted by WHO in collaboration with CDC. They have adopted this school-based methodology in collecting data. We are just using the secondary data of GSHS. There might be several disadvantages of using school-based sample which are reported in earlier studies. These includes: difficult to recruit school, increasing non-response rate, excluding students who are absent in the survey day (references below). With high nonresponse rates, the validity of population measurement is questionable. High nonresponse rate also increase the likelihood of sample bias because whole school may represent a specific segment of society. Moreover, while studying health risk behaviors using school-based sample, studies have documented that prevalence of health risk behaviors to be higher among absent respondents. These disadvantages might be considered while collecting sample.

Hallfors D, Iritani B. Local and state school-based substance use surveys—availability, content, and quality. Eval Rev. 2002;26(4):418-437.

White DA, Morris AJ, Hill KB, Bradnock G. Consent and schoolbased surveys. Br Dent J. 2007;202(12):715-717.

Thorlindsson T, Bjarnason T, Sigfusdottir ID. Individual and community processes of social closure: a study of adolescent academic achievement and alcohol use. Acta Sociol. 2007;50: 161-178.

Bovet P, Viswanathan B, Faeh D, Warren W. Comparison of smoking, drinking, and marijuana use between students present or absent on the day of a school-based survey. J Sch Health. 2006;76(4):133-137.

Michaud PA, Delbos-Piiot I, Narring F. Silent dropouts in health surveys: are nonrespondent absent teenagers different from those who participate in school-based surveys? J Adolesc Health. 1998;22(4):326-333.

However, to accommodate the disadvantage of using school-based sample, we have added the following limitation in study limitations in the discussion section (Page 15, Lines 337-339).

*“Moreover, adolescent tobacco and substance use may be underreported, because the GSHS was restricted to adolescents who attended school and were present on the day of the survey.”*

What is the proportion of adolescents in Bangladesh who do not go to school and are using tobacco?

**Response:** According to Bangladesh Demographic Health Survey 2014, the proportion of the population that do not attends school is 18% (boys: 21.7%, girls: 15%) for children age 11-15 in Bangladesh.

The 2013 Global Youth Tobacco Survey of Bangladesh reported that 6.9% of adolescents (9.2% males and 2.8% females) are currently tobacco users.

However, we have included the information of the prevalence of tobacco use in the 1^st^ paragraph of the introduction section.

How long was the self-administered questionnaire?

**Response:** The self-administered questionnaire was consisted of a total of 80 core, expanded and country-specific questions. The Bengali version of the questionnaire was used in the survey (Reference below).

Report of first Global School-based Student Health Survey (GSHS), Bangladesh, 2014. New Delhi: World Health Organization; 2018. World Health Organization, Regional Office for South-East Asia and National Centre for Control of Rheumatic Fever and Heart Disease, Ministry of Health & Family Welfare, Dhaka, Bangladesh Licence: CC BY-NC-SA 3.0 IGO.

What steps were taken by the study team to manage adolescent distress while answering questions on Multiple adverse experiences?

**Response:** To ensure privacy and confidentiality the survey was anonymous and voluntary.

Was student assent collected?

**Response:** Yes, it was. Informed consent from the students, parents, and/or school officials, as appropriate, was obtained.

We have already included this information in method section.

What language was the survey completed in and was it in the local language?

**Response:** The Bengali version of the questionnaire was used in the survey (Reference below).

Report of first Global School-based Student Health Survey (GSHS), Bangladesh, 2014. New Delhi: World Health Organization; 2018. World Health Organization, Regional Office for South-East Asia and National Centre for Control of Rheumatic Fever and Heart Disease, Ministry of Health & Family Welfare, Dhaka, Bangladesh Licence: CC BY-NC-SA 3.0 IGO.

Do participating adolescents understand and use the term marijuana colloquially?

**Response:** Marijuana is not familiar word in Bangladesh. In Bengali it is well known as *Ganja* or *weed*.

Bangladesh specific item used to measure marijuana use in the GSHS: “During the past 30 days, how many times have you used marijuana (also called ganja or weed)?”

We have corrected it in the text.

In the creation of age-standardized weights, what specific marginal population proportions were used from the population and housing census, and why? Please provide this information in a supplemental table. Or is it just based on age?

**Response:** Thank you. We used total population of the age-group 11to18 years to standardize the prevalence. We did the direct method of age-adjusted prevalence to eliminate differences in crude prevalence in populations of interest that result from differences in the populations’ age distributions. However, it is usually done when we compare two or more populations at one point in time or one population at two or more points in time. Since we are using nationally representative data, and not comparing the prevalence with other sub-group of the population; we think this age-adjusted prevalence is not necessary for the current study. Therefore, we decided to remove the age-adjusted prevalence from the table as well as from the manuscript. We think deleting these prevalence would not affect the quality of the manuscript.

See Line 160-164: Multiple imputation will not likely make the sample more representative. The citation does not match the text preceding it and it is indistinct why multiple imputation was used. It would also be useful to explain why “ever had sexual intercourse” was included in this context: “To ensure representativeness and to prevent misinterpretation or any form of biases in the analysis, the allocation of missing values was done through a multiple imputation method using a logistic regression model by taking into account the known values, most frequently for “ever had sexual intercourse” [34].”

**Response:** Thank you very much. We apologize for such mistakes. We have corrected it. Since the response of the variable “sexual history” (‘ever had sexual intercourse’ in earlier version of the manuscript) contains 11.6% missing values, we imputed these missing values. That’s why we included sexual history there. We have corrected the sentence in the following way so that it would be clearly understand by the readers (Page 9, Lines 182-185).

*“Imputation using a logistic regression model was used to estimate missing values from known values to account for missing data, most frequently for sexual history (i.e., for 11.6% of respondents). Age, gender, and school grade were included as covariates in the imputation.”*

The measures include no mention of MAE. On what basis were the measures selected? And why are they appropriate in Bangladesh? The adverse events description and combination of measures in Table 1 appear a bit arbitrary and not evidence-based. Suggestion to add a justification and description of how these measures were categorized and used based on the literature.

**Response:** Thank you. We have just followed the calculation procedure of the Adverse Childhood Experience (ACE). We just wanted to see to what extent the experience of one or more than one psychosocial or socio-environmental events are associated with substance use among adolescents. Therefore, we adopted the procedure of calculating ACE and calculated the MAE score for this study. We also inserted a paragraph describing the calculation procedure, which we indicated in responding a comment earlier. The same was followed in an earlier published article.

Khan, M.M.A., et al., *Suicidal behavior among school-going adolescents in Bangladesh: findings of the global school-based student health survey.* Social psychiatry and psychiatric epidemiology, 2020: p. 1-12. DOI: https://doi.org/10.1007/s00127-020-01867-z

**Results**

Line 175-176: Please clarify what is meant by “more than half of the adolescents had poor parental understanding?”

**Response:** Thank you. We have revised the sentence in the text.

Line 179-189: Are these differences significant, if so, please provide summary of test statistics (Confidence Intervals and p-values)?

**Response:** We have revised it in the text according to your suggestion.

Line 185 - factors than (those) who did not experience any (such factors).

**Response:** Thank you. We have corrected it in our revised manuscript.

Figure 1 does not show that boys are significantly more likely to use tobacco and substances. It only shows that the proportion of boys using tobacco is higher than girls, and only marginally so for substances.

**Response:** We have revised it accordingly.

How are the authors defining an adverse sexual history? The measures in Table 1 do not include this – it just asks, ‘Have you ever had sexual intercourse?’

**Response:** Sexual intercourse outside marriage is considered as great sin and socially unacceptable. Therefore, we defined ‘adverse sexual history’ by using the item from GSHS ‘ever had sexual intercourse’. However, the 1^st^ reviewer suggested changing the variable name ‘adverse sexual history’ to ‘sexual history’, and we did it according to him in the revised manuscript.

Line 209: The use of dose-response relationship appears inappropriate in this context as there is no experiment underway. The analysis shows an additive negative effect of adverse experiences.

**Response:** Thank you. We have changed the sentence as follows (Page 12, Lines 245-246):

*“Experience of increased number of adverse psychosocial adversities was found to be associated with greater tobacco use in a graded manner”*

Line 210-211: Why were both age and grade adjusted for as confounders?

**Response:** Thank you. While assessing the relationship of various socio-demographic, psychosocial and socio-environmental factors with tobacco and substance use using multivariable analysis, we checked the multicollinearity among the variables. We checked the value of Variance Inflation Factors (VIF), and found that in all cases the VIF values were less than 2. This indicates that multicollinearity is not a problem. Therefore, multivariable analyses were adjusted for all the socio-demographic variables included in this study. However, in some recent analyses (given below) using data from the GSHS, multivariable analyses were adjusted for both age and school grade.

Tang, J.J., et al., *Global risks of suicidal behaviours and being bullied and their association in adolescents: School-based health survey in 83 countries.* EClinicalMedicine, 2020. **19**: p. 100253.

Xi, B., et al., *Tobacco use and second-hand smoke exposure in young adolescents aged 12–15 years: data from 68 low-income and middle-income countries.* The Lancet Global Health, 2016. **4**(11): p. e795-e805.

Tables 2 should provide sample N and counts in a column on unweighted data.

**Response:** We have revised Table 2 accordingly. Thank you.

Line 220: This seems like a sentence more appropriate as an explanation in the Discussion section.

**Response:** Thank you. We have deleted that sentence from the result section and the following sentence is added in the discussion section (Page 15, Lines 324-327)

*“However, we did not find statistically significant association between experience of multiple adverse socio-environmental events and adolescents’ substance use, which could be due to very few positive responses in the multiple adverse socio-environmental categories.”*

**Discussion**

Line 227-229: Reference 26-28 are much more dated than prevalence rates reported in the Introductory paragraph.

**Response:** Although the references for prevalence of tobacco use in Bangladesh provided in the earlier version of the manuscript were published recently, they actually used data from 2013 GYTS or 2010 STEPS. We have changed those references to actual report published after those surveys in 2015 or 2011. Now those references are same.

Line 233: remove extra space before period

**Response:** Thank you. We have removed the extra space.

Line 267-270: It may be useful to bring this point earlier in the paper, maybe as a footnote to Table 1.
**Response:** Thank you for the suggestion. For the flow of the reading, we think those two sentences could be fine here as it is.

Line 275- predictors is not an accurate description of this study’s findings

**Response:** Thank you. We have revised it throughout the manuscript.

Line 280-289 and line 242-252: The idea of parental involvement/ monitoring and understanding is repetitive and may be combined effectively.

**Response:** Thank you. We have deleted some repetitive/abundant explanations from the indicated lines.

Study strengths and implications should be described in greater detail.

**Response:** Thank you. We have rewritten the strengths of this study in the text and added the following paragraph describing the implications of this study (Page 16, Lines 344-355).

*“Limitations notwithstanding, this study has the potential to inform the development and/or modification of prevention strategies. Teachers, parents or other school personnel could play important role in early identification of adolescents with these emotional and behavioral problems. However, they would require appropriate training (e.g. mental health training) and professional development opportunities to build their skills and self-efficacy to be able to recognize, and appropriately respond to the adolescents with these problems. Furthermore, the findings suggest the need for incorporating mental health education programs in school-curricula; and the importance of the family and social connections among these populations. Findings of this study underscore the importance of parental awareness to help and support adolescents in problems. Finally, since parental smoking increases the likelihood of adolescent smoking, universal prevention approaches which combine tobacco cessation campaign through mass media, including school- and community-based programs could be effective.”*

Please review:

<https://www.childwelfare.gov/topics/preventing/preventionmonth/resources/ace/>
For other descriptions of adverse childhood events and categorization.

**Response:** Thank you very much.

Line 314 – The authors themselves provide other prevalence estimates from Bangladesh, so it may seem prudent to avoid saying it is the first or even most recent?

**Response:** Thank you. We have revised it and re-written the conclusion section as follows (Page 16, Lines 357-366).

*“This study, using a nationally representative data, estimates the prevalence and risk factors associated with tobacco and substance use among school-going adolescents in Bangladesh. The findings of this study have important implications for public health professionals and practitioners who work on adolescent substance abuse programs. Several adverse psychosocial (loneliness, anxiety, bullying, and sexual history), and socio-environmental factors (parental tobacco use, lack of peer support, and poor parental monitoring) were found to be significantly associated with adolescents’ tobacco and substance use. Although the relationships are naturally complex, some patterns are discernible. Further studies with longitudinal data are needed to determine the mechanisms, processes and directionality of these relationships.”*

It may be useful to discuss mental health promotion opportunities in light of psychosocial findings.

**Response:** Thank you. We have mention about the necessity of mental health in the implication section.
