# Supplementary material for: Psychosocial and socio-environmental factors associated with adolescents’ tobacco and other substance use in Bangladesh
Source: PLoS One. 2020 Nov 24;15(11):e0242872. doi: 10.1371/journal.pone.0242872 (PMC7685447; doi:10.1371/journal.pone.0242872)
Supplement: Attachment — Submitted filename: Response to Reviewers.docx [file pone.0242872.s002.docx]

November 9, 2020

Prof. Stanton A. Glantz
Academic Editor
PLOS ONE

**Re: Manuscript Number PONE-D-20-18742R1**

Dear Prof. Stanton A. Glantz,

Thank you very much for your kind consideration of our manuscript entitled, ***“Psychosocial and socio-environmental factors associated with adolescents’ tobacco and other substance use in Bangladesh”.*** We have revised the manuscript according to the comments by the reviewers. Their useful and productive comments helped us to improve the clarity and quality of the manuscript. Where we have changed the text, the corresponding sentences in the text have been highlighted with the track changes function. The page and line numbers referred to are those in the margins of our clean revised manuscript. Where textual changes are included in this response to reviewer, we have indented and italicized them, and removed references.

We hope that the revisions are satisfactory in addressing the issues raised by the editor and the reviewers, and look forward to hearing your decision about this article.

Yours sincerely,

Md. Mosfequr Rahman, PhD

Department of Population Science and Human Resource Development

University of Rajshahi

Rajshahi-6205, Bangladesh.

E-mail: [mosfeque@ru.ac.bd](mailto:mosfeque@ru.ac.bd)

**Reviewer #1**

This was an interesting study using the latest interation of Global School-Based Student Health Survey in Bangladesh to examine association between childhood risk factors and tobacco use and substance use, which was a combined metric of alcohol and/or cannabis use. There was a big improvement from the last draft

**Response:** Thank you very much.

**Reviewer #2**

There are some minor issues that remain in the manuscript, that the authors must address:

**Response:** Thank you very much. We have addressed the issues one by one in the following sections.

1. Below table 3, the authors still use the word predictors in the manuscript- "Model was adjusted for all the predictors included in this table."

**Response:** Thank you very much. We have revised it.

2. Table 2 p-values and chi-square test values are presented in a slightly unusual format, with p-value at the base of each characteristic. Could the authors explore an alternative way of presenting whether participant characteristics are significant by tobacco users and substance users? Also, it would be useful if the authors would clarify that this table relates only to tobacco use and substance use and not tobacco use disorder or substance use disorder.

**Response:** Thank you. We have revised Table according to your suggestion. We have mentioned in both the title of the table 2 and in the text that we are assessing the prevalence of tobacco use and other substance use among the adolescents, not the disorders.

3. Use of the terms "substance abuse" remains in lines- 49, 85, 94, 121, 227, 360. If both use and abuse are being used in the manuscript, the authors may specify in the manuscript how these concepts differ and why they are both important for their study.

**Response:** We had tried to be consistent in using the term ‘use’ throughout the manuscript; however, unintentionally in some places we missed to change it. We have revised it. However, the term ‘abuse’ in line number 89 (page line of the current version) is appeared as a risk factor of substance use; this ‘abuse’ is the experience of violence. So we keep it as it was.

4. Suggestion to clarify in the manuscript - “Additionally, adolescents’ multiple adverse experience of psychosocial factors was
found to be associated with TU in a graded manner.” Perhaps the authors could say - For every additional adverse psychosocial experience there appeared to be an incremental association with increasing TU or it may work to give the exact increased odds of TU in the text. I believe the addition of MAE-relevant information in the manuscript after the initial review feedback is adequate, but in the abstract there needs to be greater clarity.

**Response:** Thank you. We have rewritten the mentioned line from the abstract as follows:

*“Additionally, higher odds of tobacco use were observed among adolescents who reported 1 (AOR: 4.36 times; 95% CI: 1.34-14.24), 2 (AOR: 8.69 95% CI: 1.67-28.23), and ≥3 (AOR: 17.46; 95% CI: 6.20-49.23) adverse psychosocial experiences than who did not report any psychosocial events.”*

5. The rationale that alcohol and marijuana are strictly prohibited in Bangladesh does not appear to justify the creation of new substance use questions when other validated measures on substance use, including both alcohol and marijuana exist. For example, the Alcohol, Smoking and Substance Involvement Screening Test (ASSIST) developed by the World Health Organization (WHO) is widely used.

**Response:** Thank you. To avoid confusion and conflict with other validated measures, and for better understanding of the readers we have change ‘substance use’ as ‘other substance use’ throughout the manuscript. Other substance use includes the use of alcohol and/or marijunana.

6. Please indicate if all 6 questions on alcohol use and all 4 questions on drug use from the GSHS were used. Please add a sentence on the use of the Bengali version of the questionnaire with its citation in the manuscript.

**Response:** Thank you. We assessed the current use (in past 30 days) of tobacco (in the form of smoking or smokeless), alcohol and/or marijuana among the adolescents. Therefore, we only used those questions (2 for current tobacco user, 1 for current alcohol use, and 1 for marijuana use) which are given in the outcome section in the text (Page Lines,) to measure the current users of these substances. We have clearly mentioned it in the outcome section.

We have added a sentence in method section (Page 7, Line 147) indicating that the Bengali version of the questionnaire was used in the survey with a reference.

7. It may be suitable for the authors to acknowledge in the manuscript that there is no existing evidence to suggest that the reasons for adolescents using tobacco in Bangladesh are similar/different from the reasons why adolescents use in global studies.

**Response:** Thank you. We have added the following sentence in the introduction (Page 6, Line 117-119)

*“Therefore, how psychosocial factors, which are found to be risk factors in some global studies, are associated with tobacco and other substance use among adolescents in Bangladesh is unknown.*”

8. Given that different tobacco prevalence studies captured varying distributions of urban-rural populations, the authors should mention what proportion of their study is an urban/ rural population in the manuscript.

**Response:** Thank you for your query. We use the secondary data from GSHS, Bangladesh, and there no information in GSHS on Rural-Urban sample. Data were not disaggregated by rural and urban. Therefore, we are unable to provide such information. However, the method section of GSHS only reported that it is a nationally representative survey.

9. It would help readers to understand the survey, if the authors would add details to the manuscript about the length of the self-administered questionnaire with references and mode (pen and pencil/ online).

**Response:** Thank you. We have added the following sentences in the method section (Page , Lines )

*“The questionnaire consisted of 80 core, expanded and country specific questions. The survey used the Bengali version of the questionnaire.”*

10. As the survey asks adolescents about multiple adverse experiences, were there any events during survey administration where participant distress was voiced/reported/observed and what did authors do as a response? Was a counsellor/ psychosocial support available? The authors should add this information to the manuscript for the benefit of readers.

**Response:** Thank you. We also think this information would benefit the readers for better understanding the survey. Since we are using secondary data from the GSHS and no such information is available in the GSHS methodology. Therefore, we are unable to include this information in the manuscript.

11. It is not clear whether student assent was collected in addition to informed consent. The authors may clarify in the manuscript.

**Response:** Thank you. We have revised the sentence as follows (Page Line 150-11). And this is the information available in the GSHS methodology.

*“To ensure voluntary participation, privacy, and confidentiality, informed consent was obtained from the students, parents and/or school officials”*

12. Please add to the measures in the manuscript why 'adverse' was a word the authors used to describe sexual history in the Bangladeshi context.

**Response:** Thank you. For addressing this issue we have added the following sentences in the text (Page 5 Line 92-101).

Earlier studies documented that early sexual initiation (i.e., sexual initiation before 16 years old) has been associated with adverse sexual health outcomes at the time of first intercourse and also later in life with an increased risk of having multiple lifetime sexual partners, unprotected sex, acquiring sexually transmitted infections (STIs), unwanted pregnancy, and undesirable sexual outcomes, such as problems with orgasm and sexual arousal. Moreover, adolescents may not be equipped to manage the psychological consequences of sexual activity; experiencing regret and having a higher risk of depression.

In addition, Bangladesh is a Muslim majority country and Islamic principles play a pivotal role in one’s life. Early and premarital sexual intercourse is not accepted and objectionable on social, religious, or moral grounds. A national survey reported that 13% of unmarried Bangladeshi male adolescents had sexual intercourse and much of this experience involves unprotected sexual intercourse with commercial sex workers. Moreover, religious, cultural and social norms are not in favor of the issue of sex education in schools, so Bangladeshi adolescents have very little knowledge on sexual and reproductive health. Bearing all these in mind, we use the term ‘averse’ while defining experience of sexual intercourse variable as ‘adverse sexual history’ in this study. However, it already creates confusion and another reviewer suggested to remove the term ‘adverse’. So we have deleted ‘adverse’. We also think global readers may get confused with the term “adverse”, so we have decided delete it. Therefore, we do not include any further description about it in the method section.
